# Changes in the Microcirculation and Physiologic Perfusion Dynamics of Free DIEP Flaps in the First 72 h After Breast Reconstruction

**DOI:** 10.3390/jcm14020520

**Published:** 2025-01-15

**Authors:** Denis Ehrl, Verena Alt, Sara Taha, Konstantin Frank, Nikolaus Wachtel, Karl J. Bodenschatz, Wolfram Demmer, Benedikt Fuchs, Riccardo E. Giunta, Nicholas Moellhoff

**Affiliations:** 1 Department for Plastic, Reconstructive and Hand Surgery, Burn Center for Severe Burn Injuries, Nuremberg Hospital, Paracelsus Medical University, Breslauer Str. 201, 90471 Nuremberg/Prof.-Ernst-Nathan Straße 1, 90419 Nuremberg, Germany; 2 Division of Hand, Plastic and Aesthetic Surgery, University Hospital, Ludwig-Maximilians-Universität München, 81377 Munich, Germany; 3 Department of Plastic, Hand and Reconstructive Surgery, University Hospital Regensburg, 93053 Regensburg, Germany; 4 Department of Pediatric Surgery and Urology, Nuremberg General Hospital, Paracelsus Medical University, 90419 Nuremberg, Germany

**Keywords:** breast reconstruction, microsurgery, autologous reconstruction, breast cancer

## Abstract

**Background/Objectives:** The autologous reconstruction of the female breast using a microsurgical DIEP flap is a reliable and safe method. To detect impairments early and preserve the microvascular flap through timely revision, a better understanding of physiologic perfusion dynamics is necessary. This exploratory study examines changes in microcirculation in free DIEP flaps within the first 72 h after vascular anastomosis using laser Doppler flowmetry and white-light spectrophotometry. **Methods:** This single-center study analyzed retro- and prospectively collected data from female patients who underwent uneventful breast reconstruction using a DIEP flap and were monitored using the O2C device (LEA Medizintechnik, Giessen, Germany). Microcirculation was monitored continuously postoperatively for a period of 72 h. **Results:** A total of 36 patients with a mean age of 48.86 (9.36) years and a mean BMI of 26.78 (4.12) kg/m^2^ received 40 DIEP flaps (four bilateral reconstructions). Microcirculatory blood flow showed a continuous increase, reaching up to 15% above its initial value within the first 72 h following anastomosis. The average tissue oxygen saturation (sO2) and relative hemoglobin (rHB) levels remained fairly stable throughout the study period, with overall reductions of 5.46% and 5.30%, respectively. **Conclusions:** Autologous breast reconstruction using a microvascular DIEP flap is a safe and reliable technique. This study showed an increase in blood flow over the 72 h study period. At the same time, sO2 and rHb showed stable levels. Deviations in these values could be interpreted as indicators of a perfusion disorder of the microvascular flap.

## 1. Introduction

Breast cancer is the most common cancer in women and the second most common cause of tumor-related death in Germany [1]. Various methods are available for reconstructing the female breast. The gold standard for breast reconstruction after mastectomy is autologous microsurgical tissue transplantation. Studies have shown higher patient satisfaction and quality of life, and success rates range up to 98% [2,3,4]. Autologous breast reconstruction using transverse rectus abdominis muscle (TRAM) flaps was first described by Holmstroem et al. in 1979 [5,6]. The Deep Inferior Epigastric Perforator (DIEP) flap commonly used today was first described 10 years later by Koshima and Soeda [7]. The high success rates of autologous breast reconstruction are achieved through the continuous development of surgical skills and techniques and microsurgical instruments, as well as the selection of a suitable patient cohort. Preoperative imaging techniques, such as CT angiography, intraoperative perfusion monitoring with indocyanine green (ICG), and meticulous postoperative monitoring, particularly of the flap, have also significantly improved outcomes [8,9,10]. The efficient postoperative monitoring of free microsurgical flaps should fulfill various requirements. Monitoring should be non-invasive and continuous. The monitoring methods must be able to quickly recognize and differentiate between venous and arterial perfusion problems and provide objectifiable results. It should be possible to monitor easily accessible flaps as well as buried flaps, and at the same time, it should be cost-effective and easy to understand for medical and non-medical staff to ensure accurate and reliable monitoring [11,12]. Clinical assessment of the perfusion and turgor of the flap as well as Doppler sonographic monitoring are standard flap monitoring measures. In addition, other methods for monitoring and the early detection of perfusion disorders, such as duplex Doppler, internal Doppler ultrasound, temperature measurements, Cook probe, pulse oximetry, microdialysis, quantitative fluorescein fluorescence, and tissue pH measurements as well as the O2C measuring device (“Oxygen to See”, LEA Medizintechnik, Giessen, Germany), are available [11,12,13,14,15,16,17,18,19]. To date, there remains an ongoing debate about the most effective type of postoperative free flap monitoring for routine clinical practice. Clinical monitoring can be susceptible to errors, as accurate assessment often depends on the experience and training of the staff responsible for postoperative monitoring. The standardized, objective, and quantifiable monitoring of microcirculation may reduce this room for error. However, microcirculatory parameters such as microvascular flow or oxygen saturation are likely to differ depending on the type of free flap, the location of the defect, the recipient vessels, and many more factors. Reference values are therefore likely to be required for each specific indication and the type of tissue transplanted. To this end, this study continuously and objectively examined the microcirculation of DIEP flaps in the first 72 h after vascular anastomosis using laser Doppler spectroscopy and white-light spectrometry with the O2C measuring device (LEA Medizintechnik, Giessen, Germany). The aim was to investigate the physiological postoperative development of perfusion in DIEP flaps in detail. The results contribute to the understanding of physiological perfusion behavior after free tissue transfer, with the aim of recognizing impaired microcirculation more quickly and effectively in the future.

## 2. Material and Methods

### 2.1. Study Design

This exploratory study examined the data of patients undergoing free DIEP flap breast reconstruction between August 2020 and December 2023. The study was approved by the Ethics Committee of the Medical Faculty of the LMU Munich (20-549).

### 2.2. Patients

**Figure 1:**
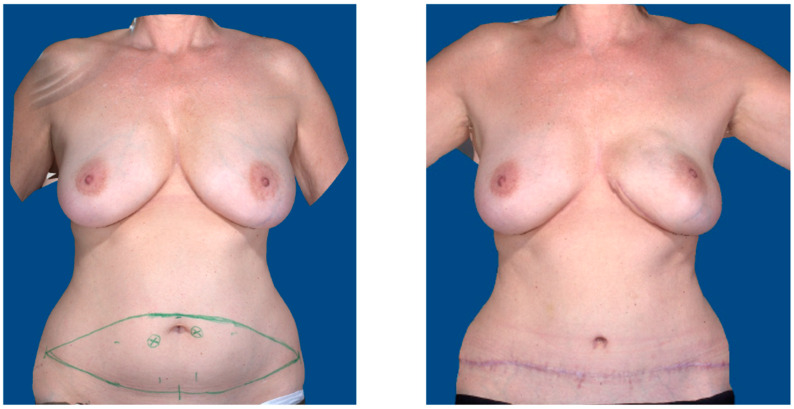
Preoperative marking (**left**) and 6-month postoperative result (**right**) of a 53-year-old patient receiving immediate breast reconstruction with a free DIEP flap after nipple-sparing mastectomy and sentinel node biopsy. Images obtained using VECTRA XT 3D imaging system (Canfield Scientific, Parsippany, NJ, USA).

All flap procedures were performed by the same lead surgeon (D.E.) to reduce any bias from the operation technique. The inclusion criteria for the study were a patient age > 18 years, uni- or bilateral breast reconstruction using a free DIEP flap (Figure 1), the ability to consent to the study, and the continuous postoperative monitoring of the flap using the O2C measuring device for up to 72 h. In order to investigate the physiological development of perfusion only, the exclusion criteria were surgical revision due to venous or arterial thrombosis, hematoma, bleeding, and major complications such as total flap loss. Thus, merely viable flaps were included. Furthermore, flaps other than DIEP flaps utilized for breast reconstruction (i.e., TMG, PAP, ms-TRAM, etc.) were excluded from analysis. Flaps that were not monitored using the O2C device (LEA Medizintechnik, Giessen, Germany) were also excluded. A total of 40 flaps performed in 36 patients (32 unilateral reconstructions and 4 bilateral reconstructions) were identified to meet the inclusion and exclusion criteria during the investigated time period.

The postoperative follow-up was standardized with additional hourly checks of the Doppler signal at the suture marking and assessments of the skin islands for turgor, color, and recapillarization time in the first 24 h, then every two hours thereafter. Monitoring was performed by qualified personnel, familiar with the process of free flap surgery. The flap was warmed using soft bandages and a warming blanket with continuous air supply. The Hb value was monitored closely and did not fall below 8 mg/dL. Postoperative fluid balancing was ensured in the first 24 h by bladder catheters and through recording the fluid intake. Patients’ source data and medical files were screened for demographics and patient characteristics.

### 2.3. Data Collection

**Figure 2:**
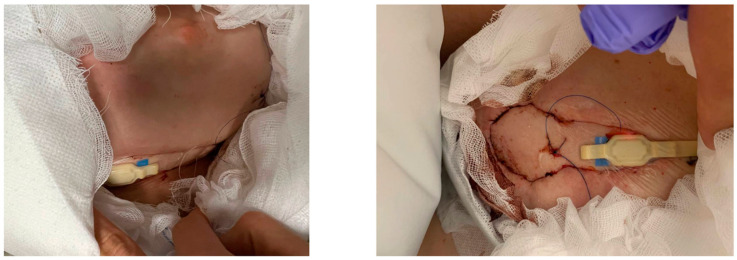
Exemplary image of the placement of the LFx37 probe of the O2C measuring device (both LEA Medizintechnik, Gießen, Germany) on the skin island of the DIEP flap in the inframammary fold (**left**) or at the position of the previous nipple–areolar complex (**right**).

The O2C device and LFx37 probe (both LEA Medizintechnik, Gießen, Germany) were used for the continuous monitoring of the DIEP flaps. The safety and reliability of the device have been proven in various studies [20,21]. Blood flow and velocity were measured by laser Doppler flowmetry, and the relative and oxygenated amount of postcapillary hemoglobin was measured by spectrophotometry at a 7 mm tissue depth (Figure 2). Data were collected continuously during the first 72 h after vascular anastomosis with constant probe attachment to the skin island of the flap. Details of the probe placement and the handling of the device have been previously described [22,23,24]. In brief, the probe was secured to the skin island of the flap using double-sided tape. The placement of the probe depended on the location of the skin island and the positioning of the flap, which varied based on the chosen point of access through the inframammary fold or a periareolar incision, as determined by the mastectomy incision.

### 2.4. Data Analysis

The data recorded with the O2C device were processed with O2CevaTime software (Version No. 28.3, LEA Medizintechnik, Giessen, Germany). The mean measured values for flow, postcapillary oxygen saturation (sO2), and relative hemoglobin (rHb) were recorded at 15 min intervals and normalized to the time of the vascular anastomoses. Hourly mean values of flow, sO2, and rHb were then extracted. The values shown are from 3, 6, 12, 24, 26, 48, 60, and 72 h post-anastomosis.

### 2.5. Statistical Analysis

The data are presented as mean values with standard deviations or as absolute and relative frequencies. All calculations were performed using SPSS Statistics 28 (IBM, Armonk, NY, USA). Normal distribution was tested using the Shapiro–Wilk test, and a probability level of *p* ≤ 0.05 was considered statistically significant.

## 3. Results

**Figure 3:**
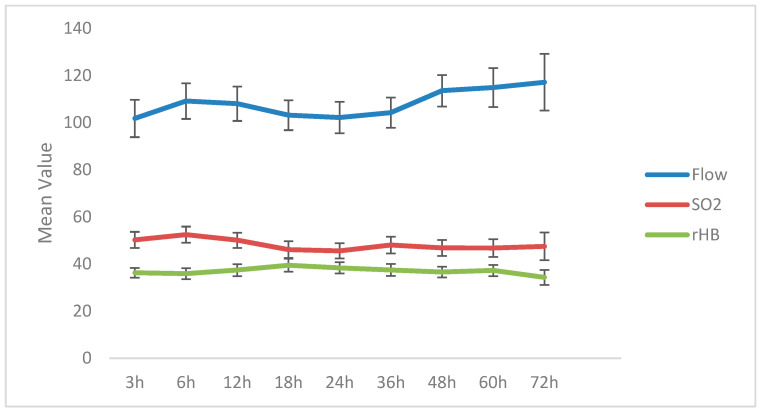
Overview of the course of microcirculation during the investigated time period.

A total of 40 DIEP flaps in 36 patients (four bilateral reconstructions) with a mean age of 48.86 (9.36) years and a mean BMI of 26.78 (4.12) kg/m^2^ were included in this study. Most patients were classified as ASA 2 (55.6%), received radiation therapy (58.3%) and chemotherapy (52.8%), and were non-smokers (83.3%). The mean ischemia time was 41.89 (7.60) minutes, and the mean hospitalization time was 7.14 (1.74) days (Table 1). The trends in microvascular flow, sO2, and rHb are summarized in Figure 3.

### 3.1. Blood Flow (Flow)

Overall, blood flow increased (+15%) over the investigated time period from a mean of 101.79 (46.25) AU at 3 h post-anastomosis to 117.16 (52.38) AU at 72 h post-anastomosis, without reaching statistical significance (*p* > 0.05) (Table 2). In detail, a modest increase was observed during the initial postoperative period, persisting for up to 6 h, after which microvascular flow stabilized with a slight decreasing trend until 36 h post-anastomosis. Following this phase, blood flow exhibited a consistent upward trajectory (Table 3).

### 3.2. Postcapillary Oxygen Saturation (sO2)

Capillary venous oxygen saturation exhibited a non-significant minimal decline over the observation period, decreasing from 50.21% (20.00) at 3 h post-anastomosis to 47.47% (25.63) at 72 h post-anastomosis (*p* > 0.05, Table 2). By the end of the 72 h monitoring period, this represented a 5.46% reduction compared to baseline levels. Overall, values remained fairly stable throughout the study period (Table 4).

### 3.3. Relative Hemoglobin (rHb)

The mean rHb level exhibited a transient increase during the initial 18 h postoperative period, followed by a subsequent decline below baseline levels (Table 5). Overall, relative hemoglobin levels did not show statistically significant changes over the 72 h postoperative period (*p* > 0.05), with the mean values for rHb being 36.24 (12.05) AU at 3 h post-anastomosis and 34.32 (13.82) at 72 h post-anastomosis (Table 2), corresponding to a reduction of 5.30%.

## 4. Discussion

The dynamic development of postoperative tissue perfusion after free DIEP flap surgery, as well as the microcirculatory changes after anastomosis, is insufficiently described in the current literature. In this exploratory study, the postoperative changes in microperfusion in DIEP flaps after autologous breast reconstruction were continuously recorded and systematically analyzed during the first 72 h postoperatively. During the study period, there was no flap loss, and a regular postoperative course was always observed in flaps monitored using the O2C device. Hence, it can be concluded that the values obtained represent the physiological perfusion dynamics of DIEP flaps. The specific development of microcirculation depends on the composition of the transferred tissue and has already been investigated in specific muscle-, fasciocutaneous and osteocutaneous free flaps [22,23,25,26]. The investigated muscle flaps showed a significant increase in blood flow after 3 days, as well as a decreasing trend line for sO2 and fairly constant rHb levels.

The data of this study show relatively constant flow behavior over the measured period. Initially, after an increase, there was a decrease in flow postoperatively. However, blood flow recovered in the further course and rose above the initial value at the end of the examination. Through consecutive measurement over a period of 48 h in 16 DIEP flaps, Figus et al. showed a continuous increase in capillary blood flow using laser Doppler flowmetry. Lightguide reflectance spectrophotometry showed first a decrease and then a continuous increase [27]. Rahamian-Schwarz et al. performed interrupted O2C measurements of DIEP flaps for up to seven days postoperatively [21]. The results differ from our continuous measurements, as they demonstrate a decrease in blood flow up until postoperative day 3, after an initial strong increase post-anastomosis.

In contrast to blood flow, our data show a slight drop in sO2 as well as in rHb during the study period. At the end of the study interval, these values were 5.46% and 5.30% below their initial values, respectively. Similarly, Rahamian-Schwarz et al. report a decrease in sO2 after anastomosis [21].

Various explanatory models can be put forward for the development of blood flow. On the one hand, the predictive power of the derived values of microcirculation is limited in the first few hours, as the autoregulation of the vascular system within the flap is compromised and minor vasospasms frequently occur. This can result in an initial decrease in blood flow [18]. In addition, surgically induced swelling can lead to increased tissue pressure, which can initially (additionally) reduce blood flow [27].

The medium-term increase in blood flow leading to a relative hyperemia of the DIEP flaps included in our study population after 72 h can be explained by the denervation of the tissue during the operation. This results in a sympathectomy of the flap, which can lead to vasodilation and reduced vascular resistance. In turn, this can result in increased blood flow within the flap [20,28]. Nasir et al. showed that the (partial) reinnervation of the flap leads to an increase in peripheral resistance after complete healing three months postoperatively [29]. Due to a change in flow behavior within the flap, there is also an increase in blood flow in the transplanted tissue. The flow velocity of the DIEP flap perforator is therefore higher than that of the recipient vessel (internal mammary artery) due to its smaller diameter (Hagen–Poiseuille law) [30,31].

Another possible explanation for the increase in flow is the intraoperative ligation of all the originally proximally draining branches of the DIEP flap [32]. The reduction in the aforementioned post-interventional swelling within the flap and the resulting decrease in peripheral resistance also favors blood flow in the flap angiosomes [28]. The technically induced intraoperative ischemia time causes hypoxic cell damage in the flap. As a result, the pH value is lowered due to the anaerobic metabolic situation. This can lead to cell death with the accumulation of anaerobic metabolites in the flap. These also lead to reactive vasodilation through the formation of nitric oxide (NO) [29,33]. The extent to which these factors persist after the restoration of perfusion through the vascular anastomoses has not been conclusively clarified. However, free oxygen radicals, which are produced as part of the xanthine oxidase and NADPH metabolic pathways after reperfusion, are particularly responsible for tissue damage in free flaps [34]. Studies have shown that an ischemia time of >60 min already leads to an increased incidence of postoperative complications and flap revisions [35]. Secondary ischemia is caused by revision surgery. A primary or secondary ischemia time > 99.5 min significantly increases the occurrence of fat necrosis [17]. The tissue composition of the flap is important for ischemia tolerance, as different tissue types have different vulnerabilities to an undersupply of oxygen. Therefore, the critical ischemia time in muscle flaps is the shortest [36,37].

The results of this study show that there was a continuous but non-significant decrease (*p* > 0.05) in capillary venous saturation (sO2) over the 72 h examination period. This decrease can be explained by the resumption of cell metabolism after ischemia-induced stasis [38]. Reperfusion leads to reactive hyperemia and increased metabolite turnover. The onset of healing and neoangiogenesis as well as the hypoxic stimulus during ischemia increases the energy and especially oxygen demand of the cells, which can cause a drop in sO2 [25,38].

Postoperative flap monitoring is of highest importance to the success of microvascular flap surgery. The continuous monitoring of perfusion parameters, as carried out in our study, can allow circulatory problems in free microvascular flaps to be recognized and differentiated at an early stage and appropriate therapy to be initiated [39].

A comprehensive understanding of microcirculatory perfusion patterns and their pathological changes contributes significantly to the timely and successful surgical revision of free flaps. The dynamics and microcirculation depend on the type of perfusion and the tissue composition of the flap. For this reason, it is particularly important to observe and track the perfusion patterns in different free flaps over a longer period of time. The O2C measurements result in individual values for different types of free flaps. The values are reproducible for the respective flap entity [38]. The data obtained in this study provide insights into the flow behavior of free flaps. The first 24 h after the creation of vascular anastomoses are considered the critical phase in which flap revision is most successful [40]. If circulatory problems are recognized early, the majority of flaps can be successfully revised [12,41,42].

Flap monitoring with an O2C device offers the possibility of continuously monitoring perfusion parameters and evaluating changes related to the respective flap type. In general, a venous outflow problem in the flap can be detected at an early stage by a continuous increase in the relative amount of hemoglobin in the flap. As the oxygen demand in the flap is increased due to the metabolic processes and tissue ischemia mentioned above, the sO2 decreases rapidly during the same period due to the lack of oxygen supply. The onset, duration, and severity of the drop in sO2 make it possible to differentiate between arterial and venous (partial) occlusions as the cause of the perfusion problem [36,37].

### Limitations

Several limitations apply to this study. First, the sample size was relatively small, which may have impacted the robustness of the findings. Additionally, the standard deviation of the data was high, which could explain the lack of statistical significance observed in the parameters of microcirculation over time.

This variability may be attributable to numerous factors beyond the type of flap reconstruction which were not accounted for in this study. Subgroup analyses are necessary to evaluate the influence of various patient characteristics, such as prior radiation therapy, smoking status, body mass index (BMI), and the number of perforators included in the flap, as well as the flap size, flap volume, and perfusion zones included within the flap [43]. To assess the individual impact of these potential confounding factors, a significantly larger sample size would be required in order to calculate a robust multivariate model. Therefore, the values presented here provide only a preliminary overview of the potential development of microcirculation in free DIEP flaps.

Potential sources of outliers in the measurements may be the insufficient shielding of the measurement area against interfering signals and the pressure applied by the measurement probe on the skin island. Individual measured values may also be falsified by manipulation, e.g., during transportation or wound checks and dressing changes, which, however, are unavoidable in a clinical setting.

Moreover, the placement of the measuring probe within the skin island, distal to the anastomosis, posed challenges. This was particularly evident in cases where most of the DIEP flap was buried, such as in nipple- and skin-sparing mastectomies, where the monitoring island was small.

## 5. Conclusions

This study examined the continuous physiologic evolution of blood flow, relative hemoglobin saturation (rHb), and postcapillary venous saturation (sO2) in DIEP flaps during the first 72 h after vascular anastomosis. The aim was to provide quantifiable reference values for the evolution of different parameters of free flap microcirculation that were measured objectively and continuously. Understanding postoperative perfusion dynamics is crucial for the postoperative evaluation of free flaps with the O2C device. Potentially, this allows plastic surgeons to recognize and address deviations and pathological developments in the perfusion of flaps at an early stage. In the future, this knowledge will further increase the success rate of microsurgical free flaps.

## Figures and Tables

**Table 1:** Demographics and patient characteristics.

	Value	
Age (years; mean, SD)	48.86	9.36
BMI (kg/m^2^; mean, SD)	26.78	4.12
ASA Status		
1	0	
2	20	
3	16	
4	0	
Radiation		
yes	21	
no	15	
Chemotherapy		
yes	19	
no	17	
Smoker		
yes	4	
never	30	
past	2	
Ischemia Time (min; mean, SD)	41.89	7.60
Hospitalization (days; mean, SD)	7.14	1.74

**Table 2:** Comparison of the mean initial (start) and final (end) measurements of all three parameters (flow, sO2, and rHb) across all analyzed DIEP flaps.

Parameter	Mean		*p*-Value
Start value: flow (AU)	101.79	46.25	
End value: flow (AU)	117.16	52.38	0.19
Start value: sO2 (%)	50.21	20.00	
End value: sO2 (%)	47.47	25.63	0.61
Start value: rHb (AU)	36.24	12.05	
End value: rHb (AU)	34.32	13.82	0.53

**Table 3:** Continuous measurement of microvascular flow over a time period of 72 h post-anastomosis.

	Flow (AU)	
Time Post-Anastomosis	Mean	SD
3	101.79	46.25
6	109.18	44.11
12	108.03	43.00
18	103.17	34.58
24	102.18	38.79
36	104.25	36.21
48	113.57	36.60
60	114.90	44.61
72	117.16	52.38

**Table 4:** Continuous measurement of postcapillary oxygen saturation (sO2) over a time period of 72 h post-anastomosis.

	sO2 (%)	
Time Post-Anastomosis	Mean	SD
3	50.21	20.00
6	52.47	19.73
12	50.09	19.23
18	46.10	19.53
24	45.59	18.66
36	48.03	20.26
48	46.83	18.68
60	46.76	20.06
72	47.47	25.63

**Table 5:** Continuous measurement of relative hemoglobin quantity (rHb) over a time period of 72 h post-anastomosis.

	rHb (AU)	
Time Post-Anastomosis	Mean	SD
3	36.24	12.05
6	35.91	13.70
12	37.40	15.02
18	39.47	15.17
24	38.35	13.91
36	37.44	14.39
48	36.57	12.50
60	37.24	12.88
72	34.32	13.82

## Data Availability

The original contributions presented in this study are included in the article. Further inquiries can be directed to the corresponding author.
